# Extramedullary Plasmacytoma of the Larynx: A Case Report of Subglottic Localization

**DOI:** 10.1155/2012/437264

**Published:** 2012-10-03

**Authors:** Jaqueline Ramírez-Anguiano, Hugo Lara-Sánchez, Deborah Martínez-Baños, Braulio Martínez-Benítez

**Affiliations:** ^1^Otolaryngology Head and Neck Surgery Service, Instituto Nacional de Ciencias Médicas y Nutrición Salvador Zubirán, Vasco de Quiroga 15, Tlalpan, 14000 Mexico City, DF, Mexico; ^2^Hematology and Oncology Department, Instituto Nacional de Ciencias Médicas y Nutrición Salvador Zubirán, Vasco de Quiroga 15, Tlalpan, 14000 Mexico City, DF, Mexico; ^3^Clinical Pathology Department, Instituto Nacional de Ciencias Médicas y Nutrición Salvador Zubirán, Vasco de Quiroga 15, Tlalpan, 14000 Mexico City, DF, Mexico

## Abstract

Extramedullary plasmacytoma (EMP) is a rare neoplasm of plasma cells, described in soft tissue outside the bone marrow. EMP of the larynx represents 0.04 to 0.45% of malignant tumors of the larynx. A male of 57 years old presented with hoarseness, dyspnea, and biphasic stridor of 2 months. The indirect laryngoscopy (IL) revealed severe edema of the posterior commissure and a polypoid mass in the right posterior lateral subglottic wall. A biopsy of the subglottic mass was performed by a direct laryngoscopy (DL). The histopathologic diagnosis was EMP CD138+, therefore radiotherapy was given at 54 Gy in 30 sessions. The patient had an adequate postoperative clinical course and a new biopsy was performed having tumor-free margins. All laryngeal lesions should be biopsied prior to treatment to determine an accurate diagnosis to guide a proper management of the condition. Radiation therapy to the EMP is considered the treatment of choice, having local control rates of 80% to 100%. The subglottis is the least accessible area of view and the least frequent location of a laryngeal mass, nevertheless the otolaryngologist should always do a complete and systematic exam of the larynx when a tumor is suspected, to detect diagnoses such as a subglottic plasmacytoma.

## 1. Introduction 

Extramedullary plasmacytoma (EMP) is a rare neoplasm of plasma cells, described in soft tissue outside the bone marrow [1]. The median age of presentation is 56–59 years [2]. It occurs predominantly in males, with a ratio of 3 : 1 [3].

The most frequently affected sites are the submucosal lymphoid tissue of the nose and sinuses [1]. It has been reported rarely in the larynx, about a 10% [3]. EMP of the larynx represents from 0.04% to 0.45% of the malignant tumors of the larynx, with an incidence less than 1% of all head and neck malignancies [4]. 

The symptoms of the extramedullary plasmacytoma are mainly dysphonia, dysphagia, cough, and dyspnea [2]. The extramedullary plasmacytomas of the larynx are usually submucosal [5].

The diagnosis of an extramedullary plasmacytoma is primarily histological, based on the presence of plasma cells which in the immunohistochemical study show monoclonality, pointing to its neoplastic nature [6, 7]. The computed tomography (CT) usually reveals a homogeneous laryngeal mass with well-defined margins, which appears with a mild-to-moderate contrast enhancement [5, 7]. Moreover, the diagnosis of extramedullary plasmacytoma is based on the exclusion of multiple myeloma [1]. 

Extramedullary plasmacytoma is a localized entity usually associated with a long surveillance [2]. Nevertheless in 16% of the cases, the disease can progress to multiple myeloma [4]. Due to the high radiosensitivity of the extramedullary plasmacytoma, the radiation alone is considered the treatment of choice [8–10]. 

## 2. Case Report

**Figure 1:**
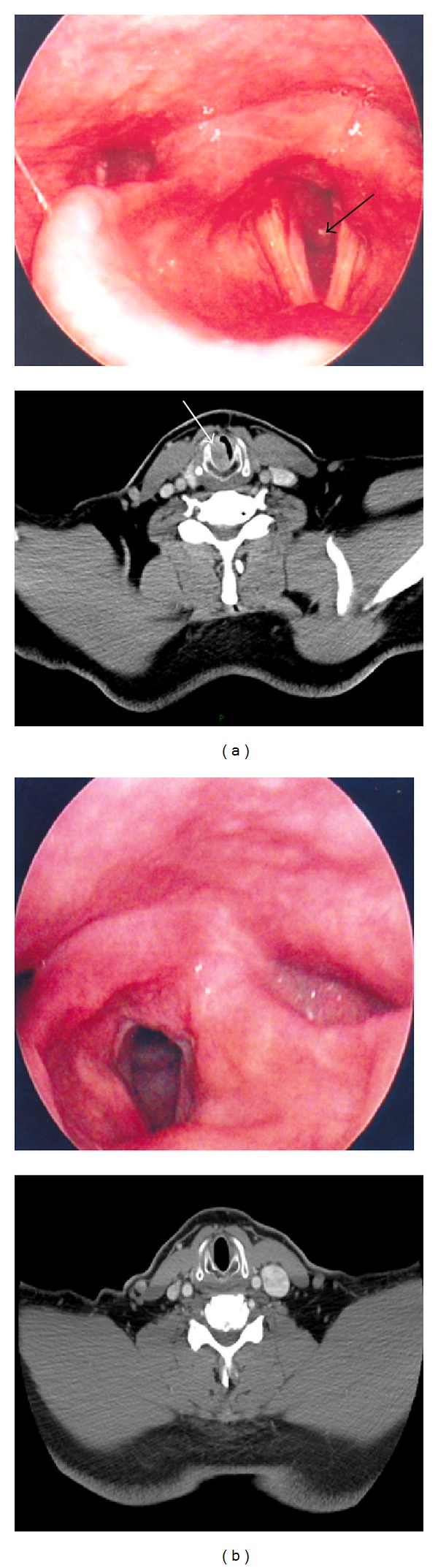
(a) At the top there is an image obtained by indirect laryngoscopy (IL) revealing severe edema of the posterior commissure and a polypoid mass on the right posterolateral subglottic wall (arrow). At the bottom there is an axial image of computer tomography (CT) at the cricoid cartilage level that shows a homogenous image with contrast enhancement at the subglottic submucosal region (arrow). (b) At the top there is an image obtained by IL 6 months after the treatment was finished. It shows absence of the subglottic mass. At the bottom there is an axial CT image at the cricoid cartilage level that reveals 100% free subglottic airway.

A male patient of 57 years old, with a history of smoking 15.5 packs a year, was diagnosed with type 2 diabetes, dyslipidemia, hyperuricemia, and gastroesophageal reflux disease. He presented to the otolaryngologist because of dysphonia, dyspnea, and a biphasic stridor of 2 months. The indirect laryngoscopy (IL) revealed severe edema in the posterior commissure of the larynx and a polypoid mass in the right posterior lateral subglottic wall (Figure 1(a)). The vocal cords were mobile with adequate glottic closure.

The neck CT image revealed a homogeneous mass reinforced by the contrast in submucosal subglottic region. It was occluding 60% of the of the subglottic airway (Figure 1(a)).

**Figure 2:**
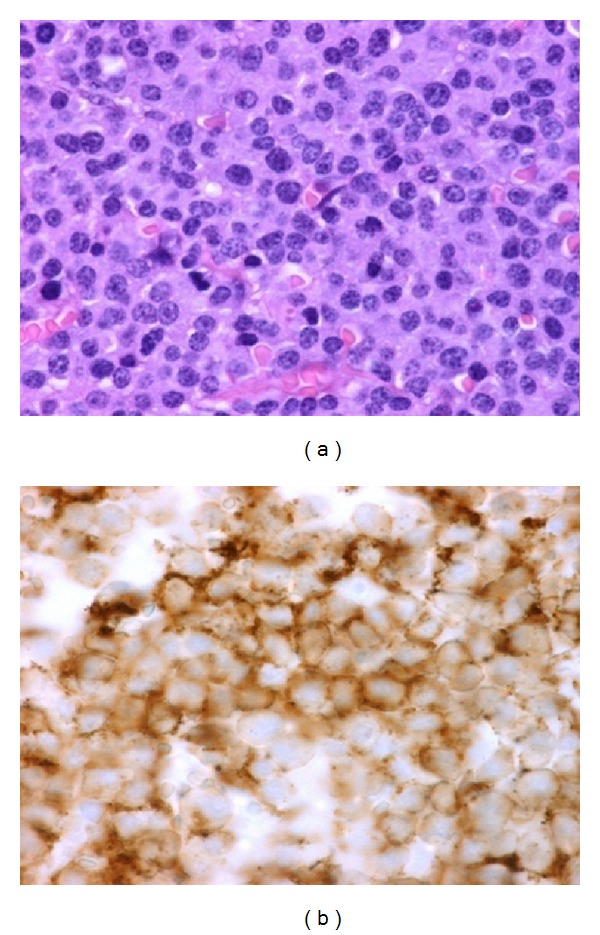
(a) Microscopic image of the submucosal subglottic portion of the mass after staining with hematoxylin and eosin. It shows intense basophilic plasma cells. (b) Microscopic image showing a positive CD138 marker at the immunohistochemical study.

The patient was hospitalized. In the operating room, a direct laryngoscopy (DL) was performed and a biopsy of the suglottic mass was obtained. The biopsy was sent to the pathology laboratory. The microscopic study revealed intense basophilic plasma cells which invade the submucosal tissue and with CD138-positive immunohistochemistry, that is characteristic of EPM (Figures 2(a) and 2(b)). The diagnosis of multiple myeloma was excluded as negative results were obtained both in the blood protein electrophoresis and urine immunofixation studies.

The patient was treated with radiation with 30 sessions of 54 Gy. The treatment was successful attaining complete remission. A month after the treatment was completed, the tumor regression was confirmed by CT having no subglottic mass and 100% free subglottic airway, and by a new biopsy with DL with free margins of the tumor. Moreover, in subsequent IL no subglottic mass at 6 months and one year after treatment was found with mobile vocal cords and an adequate glottic closure (Figure 1(b)).

## 3. Discussion

In the presence of a laryngeal mass, we must consider in the differential diagnosis an extramedullary plasmacytoma. All laryngeal lesions should be biopsied prior to treatment to determine an accurate diagnosis to guide a proper management of the condition. Radiation therapy to the extramedullary plasmacytoma is considered the treatment of choice, with local control rates of 80–100% [6].

The most common sites of presentation of laryngeal plasmacytomas are in decreasing order of frequency: the epiglottis, vocal cords, ventricular bands, the arytenoids and finally the subglottic space [5]. This case report shows the importance of the systematic examination of the three segments of the larynx: epiglottis, glottis, and subglottis. The subglottis is the least accessible area of view and the least frequent location of a laryngeal mass; nevertheless the otolaryngologist should always do a complete and systematic exam of the larynx when a tumor is suspected, to detect diagnoses such as a subglottic plasmacytoma.
